# BreakNet: detecting deletions using long reads and a deep learning approach

**DOI:** 10.1186/s12859-021-04499-5

**Published:** 2021-12-02

**Authors:** Junwei Luo, Hongyu Ding, Jiquan Shen, Haixia Zhai, Zhengjiang Wu, Chaokun Yan, Huimin Luo

**Affiliations:** 1 College of Computer Science and Technology, Henan Polytechnic University, Jiaozuo, 454003 China; 2 School of Computer Science and Information Engineering, Henan University, Kaifeng, 475001 China

## Abstract

**Background:**

Structural variations (SVs) occupy a prominent position in human genetic diversity, and deletions form an important type of SV that has been suggested to be associated with genetic diseases. Although various deletion calling methods based on long reads have been proposed, a new approach is still needed to mine features in long-read alignment information. Recently, deep learning has attracted much attention in genome analysis, and it is a promising technique for calling SVs.

**Results:**

In this paper, we propose BreakNet, a deep learning method that detects deletions by using long reads. BreakNet first extracts feature matrices from long-read alignments. Second, it uses a time-distributed convolutional neural network (CNN) to integrate and map the feature matrices to feature vectors. Third, BreakNet employs a bidirectional long short-term memory (BLSTM) model to analyse the produced set of continuous feature vectors in both the forward and backward directions. Finally, a classification module determines whether a region refers to a deletion. On real long-read sequencing datasets, we demonstrate that BreakNet outperforms Sniffles, SVIM and cuteSV in terms of their F1 scores. The source code for the proposed method is available from GitHub at https://github.com/luojunwei/BreakNet.

**Conclusions:**

Our work shows that deep learning can be combined with long reads to call deletions more effectively than existing methods.

**Supplementary Information:**

The online version contains supplementary material available at 10.1186/s12859-021-04499-5.

## Background

Genetic structural variants (SVs) normally include deletions, insertions, inversions and duplications of gene segments with variation lengths greater than 50 bp [1]. Compared to single-nucleotide polymorphisms (SNPs) and insertions and deletions (INDELs), SVs occupy a more prominent position in human genetic diversity and have a significant impact on gene functioning and gene regulation [2]. For example, the deletion of 16p11.2 (AUTS14; MIM611913) is observed for autism spectrum disorder [3]. Multiple deletions are considered responsible for schizophrenia [4], a chronic, debilitating illness that affects ~ 1% of the population. The accurate and comprehensive detection of SVs is particularly important, but the detection of SVs is much more difficult than SNP detection. Sequencing and alignment errors usually interfere with the characterization of variant regions and affect the variant detection results.

Many computational methods have been proposed. Existing methods can be divided into three categories: de novo assembly-based approaches, short-read alignment-based approaches, and long-read alignment-based approaches.

De novo assembly-based approaches can leverage both short and long reads and can use the assembled sequence or construct an assembly graph to detect SVs. De novo assembly methods can theoretically find all types of variations and are less affected by the reference sequence. However, this type of method is computationally expensive and has difficulty in terms of reconstructing haplotype sequences [5]. These shortcomings have limited the development of de novo assembly-based methods for variant detection.

Short-read alignment-based approaches directly analyse read alignments and detect SVs. Methods based on short-read sequencing technologies have been well studied; they operate by extracting SV signatures such as read depths, discordant read pairs, and split reads to find candidate sites and detect SVs [6]. Note that the signatures used in SV callers have major impacts on their performance. For example, BreakDancer relies on the distances and orientation of reads to call SVs [7], and it exhibits less sensitivity to small variations because the sizes of SVs may blend into the normal paired read distribution, and callers cannot be detected easily. Delly not only uses discordant reads as SV signatures but also takes split reads into account and shows better sensitivity to small variations [8]. LUMPY integrates discordant reads, read depths, and split reads as SV signatures to detect SVs and achieves high accuracy and sensitivity with respect to large SVs [9].

Due to technological advances and the widespread use of third-generation sequencing technologies, the greatly increased lengths of reads have helped aligners produce high-quality alignments. Longer reads can overlap better in highly repetitive or low-complexity regions that are prone to SVs [10]. Several studies have shown that long read-based methods can find a substantial number of SVs that are missed by short-read methods [11–13]. However, long reads are commonly accompanied by high sequencing error rates, and compared to that of second-generation sequencing technology, the sequencing error rate of third-generation sequencing technology is more than ten times higher. Third-generation sequencing methods also suffer from higher sequencing costs than those of short-read approaches when sequencing at the same coverage levels. Despite these disadvantages, many long read-based SV detection methods have been proposed and have achieved better performance than short read-based methods.

Sniffles is a long read-based SV caller [14]. By preforming parameter estimation at the beginning, Sniffles updates its model to fit the given data and uses a statistical model to reduce the number of false-positive calls. PBHoney is a long read-based SV detection tool designed to work with PacBio data [15]. It uses two different approaches to detect SVs. The first approach is called PBHoney-spot. By learning the stochastic natures of PacBio reads, PBHoney-spot is able to detect abnormal increases or decreases in the error rates of long reads. The second approach is PBHoney-tail. By extracting soft-clip sequences from read alignments and realigning them to the reference genome, realignments are clustered by their locations and orientations. The SV identification method (SVIM) uses both intra- and inter-alignment signatures to call SVs [16]. Intra-alignment signatures include large gaps in references and reads, and they can be extracted from CIGAR strings. Inter-alignment signatures include discordant alignment positions and abnormal read alignment orientations. The SVIM uses a graph-based cluster to separate and merge SV signatures and classify them into different classes. cuteSV uses a heuristic method to merge small SVs into large SVs, thereby producing more homogenous SV signatures. cuteSV uses a clustering-and-refinement approach to improve breakpoint accuracy and reduce the number of generated false positives [17]. The set of PacBio SV calling and analysis tools (PBSV) is an SV detection method developed by PacBio. By selecting abnormal reads and realigning them to the reference genome, PBSV exhibits better sensitivity to large insertions than other approaches.

Deep learning has gained much attention in recent years and has outperformed existing methods in many fields, such as image recognition and speech translation. In recent years, deep learning-based methods have been used by several genome variation callers [18–21]. Unlike traditional SV detection methods that rely on handcrafted designs, deep learning models can integrate information by themselves. This enables a deep learning model to find more useful information and improve SV calling. DeepVariant was the first application deep learning to the detection of SNPs; it uses short-read data to detect variants and achieves higher performance than traditional methods. DeepSV also uses short-read data with a deep learning approach, but this method focuses on large genome deletions.

However, read alignment-based SV calling still faces many difficulties. For example, due to the complexity of SVs and high sequence error rates, current methods suffer from low sensitivity to low-coverage data. Here, we present BreakNet, a deletion detection method based on long reads and deep learning. BreakNet uses a time-distributed convolutional neural network (CNN) model to extract deletion signatures and analyse the signatures with a bidirectional long short-term memory (BLSTM) model in both the forward and backward directions. BreakNet achieves better performance than existing methods and has better sensitivity to lower-coverage data.

## Methods

**Fig. 1:**
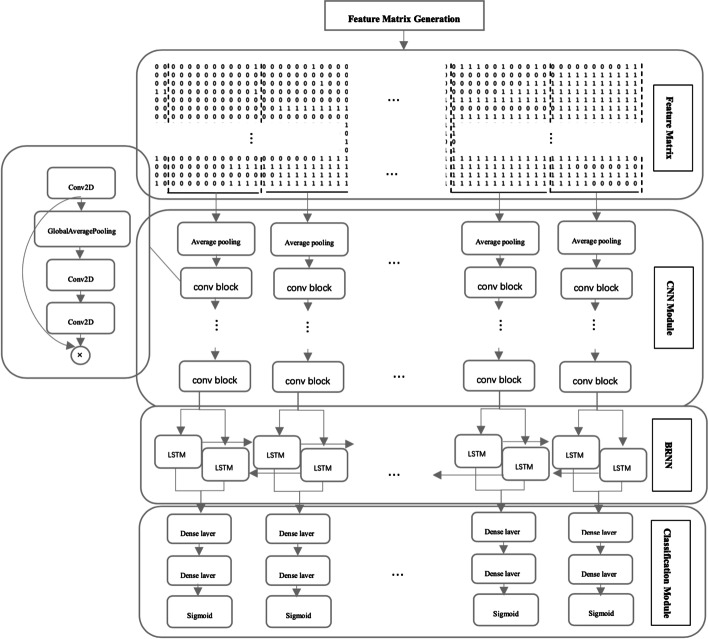
BreakNet module for detecting deletions

BreakNet uses the read alignment file produced by long reads and the corresponding reference genome as input, and it outputs deletion regions. BreakNet contains four main modules (Fig. 1). The first module is a feature matrix generation module, which splits the reference sequence into subregions, and then each subregion is transformed into a feature matrix based on alignment information. The second module is a CNN module, which maps the features to feature vectors. The third module is a bidirectional recurrent neural network (BRNN) module, which analyses the feature vectors associated with multiple time steps in both the forward and backward directions and integrates more deletion information. The fourth module is a classification module, which makes predictive judgements regarding the vectors output from the BRNN module and determines whether deletion has occurred.

### Feature matrix generation module

The feature matrix generation module first divides the reference into subregions of length *m* (200 bp by default). This module extracts long reads that can be aligned in each subregion. Then, the module extracts the CIGAR string of each aligned long read and finds the positions that refer to a deletion (D operations in a CIGAR string).

We assume that the number of aligned long reads is *p*. Then, the *i-th* aligned long read is converted to an *m-tuple* $\left( {d_{i1} , d_{i2} \cdots d_{im} } \right)$, where *d*_*ij*_ represents whether the *j-th* position in this subregion refers to a deletion based on the CIGAR string of the *i-th* aligned long read. When the *j-th* position is a deletion, *d*_*ij*_ is set to 1; otherwise, it is set to 0.

Then, these aligned long reads are sorted based on their deletion counts in descending order. Note that the numbers of aligned long reads for various sub-regions are commonly different, and the row number of each matrix is also different. To generate a matrix with the same size, we set the number of rows to *n* (18 by default). If *p* is greater than or equal to *n*, this module selects the first *n* rows to produce the matrix. Otherwise, the elements in the remaining *n – p* rows are set to 0.

### CNN module

In this module, we adopt 100 continuous transposed feature matrices as inputs. Each feature matrix is fed into a time-distributed CNN, which outputs a 160-dimensional feature vector to the next module. The CNN module consists of several operations, which include average Pooling, max pooling and 2D convolution (conv2D). In detail, this module first applies a 1 × 2 average pooling layer to downsample the input matrix and reduce the computational cost. Then, this module uses 6 convolutional blocks to map each feature matrix to the output vector. Each convolutional block consists of a conv2D layer, a squeeze-and-excitation (SE) optimization layer and a max pooling layer [22]. As an example, the convolution of a matrix can be computed by the following equation:

1 $$O_{{m^{\prime},{ }n^{\prime}}} \left[ {row,col} \right] = \mathop \sum \limits_{i = 0}^{a - 1} \mathop \sum \limits_{j = 0}^{b - 1} w_{ij} \times I_{m,n} \left[ {row + i,col + j} \right]$$

where $I_{m \times n}$ and $O_{{m^{\prime}, n^{\prime}}}$ are the input and output matrices, respectively, $w_{a*b}$ is the weight matrix of the convolution kernel, and $row, col$ are the rows and columns of the output matrix. Next, we use a rectified linear unit (*ReLU*) as the activation function to add nonlinearity.

2 $$x_{a} = ReLu\left( x \right) = \left\{ {\begin{array}{*{20}l} {0,} \hfill & {if\; m < 0} \hfill \\ {x,} \hfill & {if\; m \ge 0} \hfill \\ \end{array} } \right.$$

To enhance the performance of the network, we use SE optimization to add weights to each computed convolution channel, and the network can adaptively adjust the weight of each feature map. Similar to EfficientNet [23], the CNN module uses a 2D global average pooling layer and two 1 × 1 conv2D layers as an implementation of the SE module. Finally, we apply a max pooling operation on the weighted convolution to reduce the number of parameters.

### BRNN module

The BRNN module takes a matrix with dimensions of $\left( { T, F} \right)$ as input. *F* is set to 160, representing the 160-dimensional vector produced from the CNN module. *T* is the time step of the BRNN module, and each time step includes a hidden representation of one feature matrix. These matrices for each time step must satisfy backward and forward relations in the read alignment. In the BRNN module, we use two BLSTM layers, where each layer includes 64 LSTM units and is able to capture both the forward and reverse information about the input feature vector. The value of an LSTM cell can be calculated recursively using the following formulas.

3 $$I_{t} = sigmoid\left( {W_{I} x_{t} + U_{I} h_{t - 1} + b_{I} } \right)$$

4 $$F_{t} = sigmoid\left( {W_{f} x_{t} + U_{f} h_{t - 1} + b_{f} } \right)$$

5 $$O_{t} = sigmoid\left( {W_{O} x_{t} + U_{O} h_{t - 1} + b_{O} } \right)$$

6 $$C_{t} = F_{t} C_{t - 1} + I_{t} \odot {\text{tanh}}\left( {W_{C} x_{t} + U_{C} h_{t - 1} + b_{C} } \right)$$

7 $$h_{t} = O_{t} \odot tanh\left( {C_{t} } \right)$$

$I,F,O$ represent the activation vectors of the input gate, forget gate and output gates, respectively. $C$ represents the state vector of the cell. $W, U, b$ are the parameters of the LSTM cell.$x,h$ are the input and output vectors of the LSTM cell, respectively. The output vector of an LSTM cell depends on the current input vector, the output vector of the hidden layer at the previous time step and the information stored in the LSTM cell.

### Classification module

The classification module uses two fully connected layers to classify the vectors output by the BRNN module. Dropout is used after each fully connected layer to improve the generality of the network. Finally, a sigmoid function is used to calculate the output values of the fully connected layers. If the final output is greater than 0.5, the related subregion is a deletion; otherwise, this subregion is not a deletion.

### Breakpoint estimation

For a deletion whose size is larger than the window size, the deletion will present in multiple subregions and corresponds to multiple feature matrices. Then these adjacent sub-regions will be merged as a large region which refers to a large deletion.

Next, for each region which is determined as a deletion, BreakNet will assign a more precise deletion location and size. For a long read which is aligned in this region, BreakNet extracts its’ deletion location and size from CIGAR string if its’ deletion size is larger than 20 bp. After processing all these long reads, BreakNet can get a deletion set {(*L*_*1*_*, S*_*1*_), (*L*_*2*_*, S*_*2*_), … (*L*_*t*_*, S*_*t*_)}, *L*_*i*_ and *S*_*i*_ separately represent the location and size of *i*-th deletion information from CIGAR strings. Then, BreakNet will classify (*L*_*i*_*, S*_*i*_) and (*L*_*j*_*, S*_*j*_) into the same cluster, if |*L*_*i*_* – L*_*j*_| < 40. After iteratively processing all elements in the deletion set, BreakNet selects the cluster which contains the most elements. And BreakNet calculates the average location and size of all elements in the cluster, which are the final location and size of the deletion about the region.

### Model training

Through the high-confidence call set of one sample, we determine the real deletion regions in the sample. Then, we label the subregions that overlap with the real deletion as 1, while the rest are labelled as 0. The model learns its parameters by minimizing a loss function.

However, the high-confidence call set used in this study has two key features. One is that the information provided in the call set may not be comprehensive, and some of the variances are not provided in this call set. Second, the information provided in the call set is relatively accurate, and there are few false positives. Therefore, based on the above characteristics, we design a new loss function to satisfy the following requirements. First, if a deletion predicted by the model is not provided by the high-confidence call set, the loss function should produce a smaller loss value and gradient. Second, if a deletion is provided by the high-confidence call set and not detected by this model, the loss function should yield a larger loss value and gradient.

8 $$\ln \left( {prediction - label + 1 + a} \right)^{2}$$

where $prediction$ is the output value of the model,$label$ is the label of the input data point and $a$ is a hypermeter used to adjust the maximum output value of this loss function. We set $a$ to 0.001 in this paper.

Because deletions are usually rare, ~ 99% of the training samples are negative. To ensure that sufficient positive samples are included at each training step, we first adequately shuffle the training data and use a large batch size. We use the Adam optimizer for training and set the learning rate to 0.001.

Two techniques are employed to prevent overfitting during the model training process. First, we add dropout layers to each fully connected layer and set the dropout probability to 0.4. Second, we use the early stopping technique during training. By examining the performance of the model on the validation set, we save the parameters with the best performance. Additionally, if the model does not perform better after 10 epochs, we stop the training process and save the best model parameters as the final model.

We use TensorFlow 2.4 to implement the CNN, BRNN, and classification modules.

## Results

### Data set

In this paper we used several read alignment files from four well studied individuals, HG002, HG00514, HG00733 and NA19240. The details of these datasets are shown in Table 1.

**Table 1:** The detail of datasets

	HG002 CLR	HG002 CCS	HG00514	HG00733	NA19240
Read count	29,157,344	6,596,012	12,430,587	13,521,896	20,452,822
Average length	7937	13,478	11,800	12,295	6503
Coverage	69X	28X	42X	45X	39X
Aligner	NGMLR	PBMM2	BWA	BWA	BWA

We benchmarked BreakNet, SVIM (v2.0.0), cuteSV (v1.0.6) and Sniffles (v1.0.12a) with several real sequencing datasets. Four PacBio datasets (HG002 CLR, HG002 CCS, HG00514 CLR, and HG00733 CLR) were used. The support read parameters of the SVIM, cuteSV and Sniffles were set to 10/4/4/3 and 3/2 for the HG002 CLR 69X, 35X, 20X, and 10X datasets and the HG002 CCS 28X and 10X datasets, respectively. For the HG00514 and HG00733 data, we set the support read parameters of Sniffles, cuteSV, and the SVIM to 3/2/1. Deletion sizes smaller than 50 bp were removed. Truvari (v2.0.1) was used to obtain the evaluation metrics (precision, recall, and F1 score) and assess the performance of the different deletion callers.

For the HG00514 and HG00733 samples, we collected a call set from a previous study [24] and considered it the ground truth. For the HG002 dataset, two deletion call sets, Tier 1 and Tier 2, for this sample (made by the Genome in a Bottle Consortium (GIAB)) were used. According to the GIAB [25], the Tier 1 region should include 100% true deletions, and we used the Tier 1 region to assess the precision, recall and F1 score of each caller. The Tier 2 regions were defined as additional regions in which there was strong evidence regarding the presence of an SV, but the corresponding sequence and size could not be determined with confidence [25]. This may be useful for benchmarking a tool’s ability to detect more challenging SVs’. The results of the performance comparison for the HG002 Tier 2 region are provided in the supplementary materials. Note that, GIAB released high quality result (~ 90%) comes from PBSV. To be fair, the callers are not compared with PBSV.

### Data partition for BreakNet

We partition the dataset into a training set, test set and validation set. The read alignments of chromosomes 1–10 from the HG002 PacBio Continuous Long Reads (CLR), HG002 PacBio Circular Consensus Sequencing (CCS) and NA19240 PacBio CLR data are used as the training set. The read alignment files of HG002 and NA19240 are generated by the NGMLR aligner, pbmm2 aligner and Burrows-Wheeler aligner (BWA). By training the model using the data produced by different aligners, the model can adapt to the varying characteristics of the different alignment tools. The validation set is used to provide an unbiased estimate of the model's performance, and this set is not directly involved in the training process. By analysing the performance of the model on the validation set and tuning the hyperparameters during training, the validation set can help to improve the performance of the model and prevent overfitting. In this paper, the read alignments of chromosome 11 from HG002 and NA19240 are used as the validation set.

For the test set, we use the read alignments of chromosomes 12–22 from the HG002 CCS and CLR data and the read alignments of chromosomes 1–22 from the HG00514 and HG00733 data as the test set. The read alignments of HG002 CLR and HG002 CCS and HG00514 and HG00733 are produced by the NGMLR, pbmm2 and BWA aligners, respectively.

### SV detection results on the HG002 dataset

We benchmarked BreakNet, cuteSV, the SVIM and Sniffles on the HG002 CLR 69X chromosome 12–22 data. The SVIM achieved the highest F1 scores and recall on the Tier 1 region. On the more challenging Tier 2 region, BreakNet outperformed the other callers in terms of recall by 3–4%. Furthermore, we randomly downsampled the HG002 chromosome 12–22 data to 10 × , 20 × , and 35 × to assess the capabilities of the SV callers on low-coverage datasets. As shown in Table 2, BreakNet achieved higher F1 scores on the 10X and 20X downsampled Tier 1 regions. This demonstrated that BreakNet is a better option for cost-sensitive sequencing plans (low coverage). For the low-coverage Tier 2 region, the highest sensitivity was obtained by BreakNet with respect to more challenge deletions.

**Table 2:** Performance comparison of SV caller on HG002 data

	Coverage		BreakNet	SVIM	cuteSV	SNIFFLES
CLR	69X	Precision	0.9704	0.9678	**0.9707**	0.9604
		Recalll	0.9169	**0.9341**	0.9282	0.9224
		F1	0.9429	**0.9507**	0.9492	0.9410
	35X	Precision	0.9469	0.9653	**0.9775**	0.9556
		Recall	0.9169	0.9292	0.8955	0.9160
		F1	0.9316	**0.9468**	0.9351	0.9355
	20X	Precision	0.9524	**0.9722**	0.9790	0.9720
		Recall	**0.8776**	0.8389	0.8203	0.7983
		F1	**0.9135**	0.9004	0.8926	0.8770
	10X	Precision	0.9213	**0.9790**	0.9819	0.9785
		Recall	**0.8134**	0.6704	0.6646	0.6470
		F1	**0.8640**	0.7959	0.7925	0.7790
CCS	28X	Precision	0.9552	**0.9400**	0.9492	0.9020
		Recall	0.9350	0.9430	0.9336	0.8325
		F1	**0.9450**	0.9415	0.9414	0.8657
	10X	Precision	0.9424	**0.9360**	0.9609	0.9110
		Recall	0.9282	0.8940	0.8398	0.6357
		F1	**0.9353**	0.9146	0.8965	0.7490

Bold values represent best results

For PacBio CCS chromosomes 12–22 in the 28X Tier 1 region, BreakNet achieved better F1 scores, which were 0.35% higher than those of the runner-up method (the SVIM). Furthermore, we randomly downsampled this dataset to 10 × and evaluated the compared callers. BreakNet achieved the highest F1 scores and lowest performance loss (~ 1% vs. ~ 3 ~ 11%) in comparison with to the other callers. The results of the performance comparison among the various callers for the Tier 2 region are provided in the supplementary materials (Additional file 1: Table S1).

### SV detection results on the HG00514 and HG00733 datasets

We used two other PacBio CLR datasets from two well-studied human samples (HG00514 and HG00733) to benchmark the compared SV callers. BreakNet simultaneously achieved the highest precision, recall and F1 scores on these two datasets, as shown in Table 3. Compared to the runner-up method (Sniffles), BreakNet achieved performance improvements of ~ 4–6% in terms of the F1 scores; this was mainly due to the higher sensitivity of BreakNet. Sniffles achieved better performance than to cuteSV and the SVIM with respect to the precision and F1 scores. Furthermore, we randomly downsampled these two datasets to 24X/21X and 10X and analysed the performance of various callers on the corresponding low-coverage datasets. BreakNet achieved the best performance on these datasets in terms of the recall and F1 score metrics. Sniffles attained the best overall precision on these four downsampled datasets.

**Table 3:** Performance comparison of SV caller on HG00514 and HG00733 data

			BreakNet	Sniffles	cuteSV	SVIM
HG00514	42X	Precision	**0.7197**	0.6772	0.4539	0.5547
		Recall	**0.351**	0.3137	0.3286	0.2261
		F1	**0.4721**	0.4290	0.3811	0.3213
	21X	Precision	0.5900	**0.7358**	0.4660	0.4407
		Recall	0.2624	0.2898	**0.3442**	0.1632
		F1	0.3633	**0.4158**	0.3960	0.2382
	10X	Precision	0.5552	**0.5986**	0.5562	0.2593
		Recall	**0.3310**	0.2585	0.3013	0.3003
		F1	**0.4147**	0.3611	0.3909	0.2783
HG00733	48X	Precision	**0.7160**	0.6528	0.4834	0.5166
		Recall	**0.3578**	0.3076	0.2932	0.2277
		F1	**0.4772**	0.4182	0.3650	0.3162
	24X	Precision	**0.7598**	0.7197	0.4250	0.5981
		Recall	**0.3386**	0.2832	0.3379	0.2218
		F1	**0.4685**	0.4065	0.3765	0.3235
	10X	Precision	0.5444	**0.6440**	0.5117	0.2361
		Recall	**0.3364**	0.2510	0.2959	0.3147
		F1	**0.4158**	0.3611	0.375	0.2700

Bold values represent best results

### Window size and deletion size

For verifying the influence of the widow size on the detection results, we separately set the widow size to 50 bp, 100 bp, 200 bp, 400 bp, 800 bp and run BreadNet. The HG002 CLR 69X chromosome 1 and chromosome 2 are used to generate training and test data separately. The training and test results are shown in the supplementary materials (Additional file 1: Fig. S1).

Considering the performance of each caller with different deletion sizes, BreakNet also can achieve better performance for deletion sizes below 4000 bp, especially on the low-coverage data. The results of the performance comparison among the various callers with different deletion sizes are provided in the supplementary materials (Additional file 1: Fig. S2).

### Loss function

**Fig. 2:**
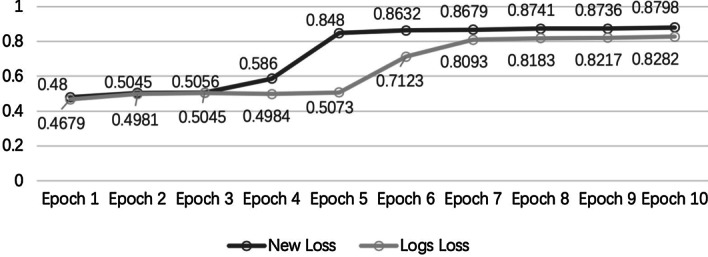
Effect of using new loss and log loss functions on the AUC values of the model training

To verify the effectiveness of our proposed loss function, we trained BreakNet with the new loss function (Formula 8) and the log loss function. Training the model with the new loss function was faster and yielded a higher area under the receiver operating characteristic curve (AUC) during each training epoch. As shown in Fig. 2, the new loss function used only five training epochs and achieved better AUC values than those output by the log loss function with ten training epochs (0.848 vs. 0.828). Because the training data involved a class-imbalanced dataset, the new loss function produced larger gradients for data that were incorrectly predicted to be negative and helped the model complete training faster. Simultaneously, the large gradients produced by the new loss function also reduced the impact of incorrectly labelled data on model training.

### Running time and peak memory

We perform all callers on a PC with a 64-core, 128-thread CPU (AMD Ryzen Threadripper 3990X @4.8 GHz). We use a single RTX 3090 video card to train the model. The running time and peak memory usage of BreakNet on HG002 are provided in the supplementary materials (Additional file 1: Table S2 and Additional file 1: Table S3). As shown in Additional file 1: Table S2 and Additional file 1: Table S3, the efficiency of BreakNet has a poor performance. BreakNet needs to generate lots of feature matrices, which consumes more resource. Note that, the training time of BreakNet is time consuming (~ 30 days). We supply the module that has been trained for users. And the users only need generate the feature matrices and could apply the trained module to obtain the detection results. In Additional file 1: Table S2, we ignore the training time. For the same species, no matter the sequencing coverage, the number of feature matrices is the same for BreakNet. Hence, the peak memory usage of BreakNet varies little.

## Discussion

Long-read sequencing technologies are promising for discovering SVs. Due to the high sequencing errors and the complexity of SVs, it is still nontrivial to fully take advantage of long-read technologies. In this study, we developed BreakNet to detect deletions based on a deep learning method and long reads. We tested BreakNet on several well-studied datasets, and it could achieve better performance than three other SV callers. However, long-read sequencing still incurs a greater computational cost than short-read sequencing when the same coverage is required. Previously developed methods require high data coverage to detect sensitive call deletions, and low-coverage data have a great impact on sensitivity. BreakNet achieved more stable performance than the other approaches on low-coverage data, especially on the HG002 CLR 10X dataset. BreakNet outperformed the runner-up method (the SVIM) by 6%.

In this paper, we only took deletions into consideration, but other types of variations, such as insertions, inversions, and copy number variations, play important roles in human health. We will examine the calling of other types of SVs in future work.

## Conclusions

In this paper, we present BreakNet, which is a deletion detection method that utilizes long reads and deep learning. Compared to state-of-the-art SV callers, BreakNet yields better F1 scores on most datasets and provides better sensitivity and F1 scores on low-coverage data. Limited by the features extracted by BreakNet, only deletions can be efficiently detected. The extraction of more features to call other types of SVs will be investigated in our future work.

## Supplementary Information

**Additional file 1.**

## Data Availability

The HG002 data can be downloaded from https:// ftp.ncbi.nih.gov/giab/ftp/data/AshkenazimTrio. The high confidence callset and the high confidence regions of HG002 were provided by GIAB and downloaded from https://ftp-trace.ncbi.nlm.nih.gov/giab/ftp/data/AshkenazimTrio/analysis/NISTSVsIntegrationv0.6/HG002SVsTier1v0.6.vcf.gz, https://ftp-trace.ncbi.nlm.nih.gov/giab/ftp/data/AshkenazimTrio/analysis/NIST SVs Integrationv0.6/HG002SVs Tier1v0.6.bed. The alignment files of samples HG00514, HG00733 and NA19240 can be downloaded from ftp://ftp.1000genomes.ebi.ac.uk/vol1/ftp/datacollections/hgsvsvdiscovery/working/20160905smithmpacbioaligns/. The high confidence callset for these three indi- viduals are collected from previous study [22], and download from NCBI dbVAR: ftp://ftp.ncbi.nlm.nih.gov/pub/dbVar/data/Homosapiens/bystudy/vcf/n-std152.GRCh37.variantcall.vcf.gz. The software and sample result as part of this project are readily avail- able from GitHub at https://github.com/luojunwei/BreakNet.

## Abbreviations

BAM: Binary sequence alignment
CNN: Convolutional neural network
BRNN: Bidirectional recurrent neural networks
SNPs: Single nucleotide polymorphisms
INDELs: Small insertion and/or deletion
SVs: Structural variations
