# Supplementary material for: BreakNet: detecting deletions using long reads and a deep learning approach
Source: BMC Bioinformatics. 2021 Dec 2;22:577. doi: 10.1186/s12859-021-04499-5 (PMC8641175; doi:10.1186/s12859-021-04499-5)
Supplement: Supplementary file 1 — Additional file 1. [file 12859_2021_4499_MOESM1_ESM.doc]

**Supplementary Materials**

| **Table S1. Performance comparison of SV caller on HG002 Tier1, Tier2 region** | | | | | | | |
| --- | --- | --- | --- | --- | --- | --- | --- |
| **Coverage** | **Callers** | **Precision_Tier1** | **Recall_Tier1** | **F1_Tier1** | **Precision_Tier2** | **Recall_Tier2** | **F1_Tier2** |
| **69X CLR** | Breaknet | 0.9704 | 0.9169 | 0.9429 | 0.9368 | **0.7803** | **0.8514** |
|  | SVIM | 0.9678 | **0.9341** | **0.9507** | **0.9697** | 0.7365 | 0.8372 |
|  | cuteSV | **0.9707** | 0.9282 | 0.9492 | 0.9575 | 0.7391 | 0.8343 |
|  | Sniffles | 0.9604 | 0.9224 | 0.9410 | 0.9608 | 0.7532 | 0.8444 |
| **35X CLR** | Breaknet | 0.9469 | 0.9169 | 0.9316 | 0.9376 | **0.7715** | **0.8465** |
|  | SVIM | 0.9653 | **0.9292** | **0.9468** | 0.9643 | 0.735 | 0.8342 |
|  | cuteSV | **0.9775** | 0.8955 | 0.9351 | 0.9657 | 0.7224 | 0.8265 |
|  | Sniffles | 0.9556 | 0.9160 | 0.9355 | **0.9676** | 0.7266 | 0.83 |
| **20X CLR** | Breaknet | 0.9524 | **0.8776** | **0.9135** | 0.9367 | **0.7589** | **0.8385** |
|  | SVIM | 0.9722 | 0.8389 | 0.9004 | 0.977 | 0.6306 | 0.7665 |
|  | cuteSV | **0.9790** | 0.8203 | 0.8926 | 0.9732 | 0.6291 | 0.7642 |
|  | Sniffles | 0.9720 | 0.7983 | 0.8770 | **0.9779** | 0.579 | 0.7273 |
| **10X CLR** | Breaknet | 0.9213 | **0.8134** | **0.864** | 0.9541 | **0.6844** | **0.797** |
|  | SVIM | 0.9790 | 0.6704 | 0.7959 | 0.9712 | 0.4908 | 0.6521 |
|  | cuteSV | **0.9819** | 0.6646 | 0.7925 | 0.9698 | 0.4788 | 0.6411 |
|  | Sniffles | 0.9785 | 0.6470 | 0.7790 | **0.9759** | 0.4329 | 0.5998 |
| **28X CCS** | Breaknet | **0.9552** | 0.935 | **0.945** | 0.9129 | 0.7772 | 0.8396 |
|  | SVIM | 0.9400 | **0.9430** | 0.9415 | **0.9295** | **0.7934** | **0.8561** |
|  | cuteSV | 0.9492 | 0.9336 | 0.9414 | 0.8674 | 0.7652 | 0.8131 |
|  | Sniffles | 0.9020 | 0.8325 | 0.8657 | 0.8711 | 0.6828 | 0.7655 |
| **10X CCS** | Breaknet | 0.9424 | **0.9282** | **0.9353** | 0.886 | **0.7751** | 0.8269 |
|  | SVIM | 0.9360 | 0.8940 | 0.9146 | **0.8991** | 0.7736 | **0.8316** |
|  | cuteSV | **0.9609** | 0.8398 | 0.8965 | 0.8891 | 0.7386 | 0.8069 |
|  | Sniffles | 0.9110 | 0.6357 | 0.7490 | 0.8559 | 0.4783 | 0.6137 |

For verifying the influence of the widow size on the detection results, we separately set the widow size to 50bp, 100bp, 200bp, 400bp, 800bp and run BreadNet. The HG002 CLR 69X chromosome 1 and chromosome 2 are used to generate training and validation data separately. The training and validation results are shown in Figure S1.

As shown in Figure S1, the detection results with the window size 50bp and 100bp have poor performance both in precision and recall. And the performance on validation set is unstable and more difficult to train. Larger window size (200bp, 400bp, 800bp) shows smoother curve in both training and validation datasets, and higher precision and recall are achieved. When setting window size to 400bp, the best performance is achieved.


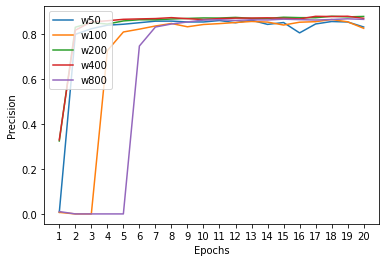

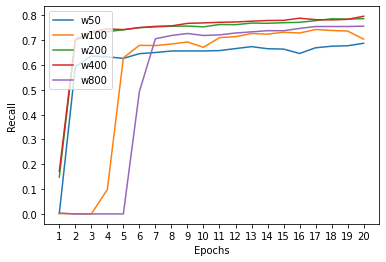


(a) (b)


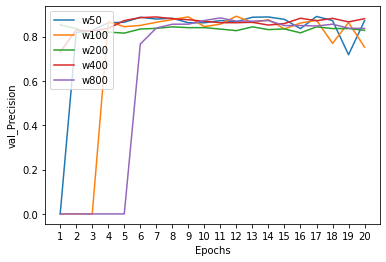

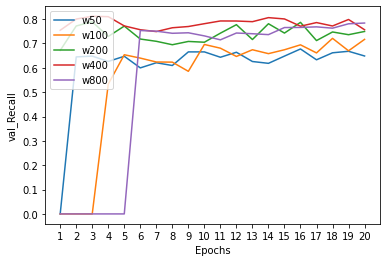


(c) (d)

Figure S1 The performance impact of different window size. (a) The precision of different window sizes on training data. (b) The recall of different window sizes on training data. (c) The precision of different window sizes on validation data. (d) The recall of different window sizes on validation data.

Table S2 The running time of different callers on HG002 CLR/CCS data

|  | Breaknet (m) | SVIM (m) | cuteSV (m) | SNIFFLES (m) |
| --- | --- | --- | --- | --- |
| HG002 CLR 69X | 1,049 | 182 | 418 | 121 |
| HG002 CLR 35X | 705 | 80 | 215 | 99 |
| HG002 CLR 20X | 374 | 39 | 108 | 46 |
| HG002 CLR 10X | 232 | 19 | 55 | 23 |
| HG002 CCS 28X | 479 | 23 | 48 | 57 |
| HG002 CCS 10X | 234 | 9 | 14 | 17 |

Table S3 The peak memory usage of different callers on HG002 CLR/CCS data

|  | Breaknet (kb) | SVIM (kb) | cuteSV (kb) | SNIFFLES (kb) |
| --- | --- | --- | --- | --- |
| HG002 CLR 69X | 42,485,976 | 11,474,980 | 278,628 | 22,322,728 |
| HG002 CLR 35X | 42,517,836 | 6,024,736 | 169,448 | 12,508,564 |
| HG002 CLR 20X | 42,398,396 | 3,114,884 | 104,904 | 5,737,184 |
| HG002 CLR 10X | 42,153,768 | 1,637,448 | 73,976 | 2,772,664 |
| HG002 CCS 28X | 41,830,036 | 688,544 | 88,260 | 1,687,264 |
| HG002 CCS 10X | 34,913,676 | 298,640 | 82,800 | 550,368 |


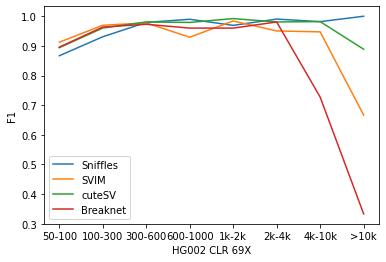

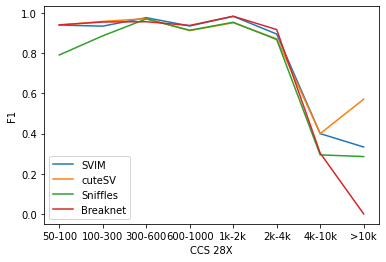


(a) (b)


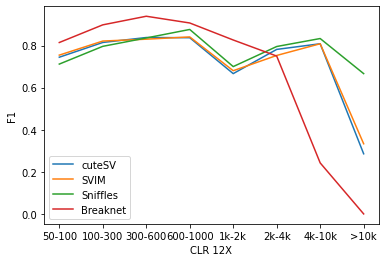

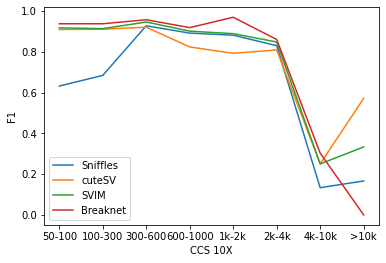


(c) (d)

Figure S2 The performance comparison on different deletion sizes of each caller. (a) The F1-score of different deletion sizes on HG002 69X CLR data. (b) The F1-score of different deletion sizes on HG002 28X CCS data. (c) The F1-score of different deletion sizes on HG002 12X CLR data. (d) The F1-score of different deletion sizes on HG002 10X CCS data.
